# Relationship Between Depression and Falls Among Nursing Home Residents: Integrative Review

**DOI:** 10.2196/57050

**Published:** 2024-11-28

**Authors:** Alcina Matos Queirós, Armin von Gunten, Joëlle Rosselet Amoussou, Andreia Maria Lima, Maria Manuela Martins, Henk Verloo

**Affiliations:** 1 Departement of Health and Social Welfare Lausanne University Hospital and University of Lausanne Lausanne Switzerland; 2 Institute of Biomedical Sciences Abel Salazar University of Porto Porto Portugal; 3 Service of Old Age Psychiatry Lausanne University Hospital and University of Lausanne Lausanne Switzerland; 4 Medical Library-Cery Lausanne University Hospital and University of Lausanne Prilly Switzerland; 5 Polytechnic Institute of Viana do Castelo Viana do Castelo Portugal; 6 School of Nursing Sciences University of Applied Sciences Western Switzerland Sion Switzerland

**Keywords:** depression, falls, nursing home, nursing home resident, cross-sectional study, cohort study, integrative review, fall risk, older adults

## Abstract

**Background:**

Depression is a highly prevalent psychopathological condition among older adults, particularly those institutionalized in nursing homes (NHs). Unfortunately, it is poorly identified and diagnosed. NH residents are twice as likely to fall as community-dwelling older adults. There is a need for more knowledge about the mechanisms and relationships between depression and falls.

**Objective:**

This study aims to identify, analyze, and synthesize research on the relationships between depression and falls among NH residents.

**Methods:**

A literature search was conducted in October 2023 in the following bibliographic databases: MEDLINE ALL Ovid, Embase, CINAHL with Full Text EBSCO, APA PsycInfo Ovid, Web of Science Core Collection, the Cochrane Database of Systematic Reviews Wiley, and ProQuest Dissertations & Theses A&I. Clinical trials were searched for in the Cochrane Central Register of Controlled Trials Wiley, ClinicalTrials.gov, and the World Health Organization International Clinical Trials Registry Platform. Additional searches were performed using Google Scholar, the DART-Europe E-theses Portal, and backward citation tracking. The Newcastle-Ottawa Scale and the Appraisal tool for Cross-Sectional Studies were used to evaluate study quality.

**Results:**

The review included 7 quantitative studies published in 7 different countries from 3 continents; of these, 6 (86%) were cross-sectional studies, and 1 (14%) was a prospective cohort study. Results suggested high frequencies of depressive symptoms and falls among older adults living in NHs, and depressive symptoms were considered a risk factor for falls. The 15-item and 10-item versions of the Geriatric Depression Scale were the most commonly used measurement tools, followed by the Cornell Scale for Depression in Dementia and the Resident Assessment Instrument-Minimum Data Set 2.0. The prevalence of depression was heterogeneous, varying from 21.5% to 47.7% of NH residents. The studies used heterogeneous descriptions of a fall, and some considered the risk of falls, recurrent fallers, and near falls in their data. The prevalence of fallers was disparate, varying from 17.2% to 63.1%. Of the 7 retained studies, 6 (86%) reported a relationship between depression and falls or the risk of falls. Among the 19 other risk factors identified in the review as being associated with falls among NH residents were a history of falls in the last 180 days, >1 fall in the past 12 months, and respiratory illnesses.

**Conclusions:**

There is a paucity of research examining falls among older adults with depressive symptoms in NHs. These findings should alert nurses to the need to consider depression as a risk factor in their work to prevent falls. More research is needed to gain a comprehensive understanding of fall risk among NH residents with depressive symptoms.

**International Registered Report Identifier (IRRID):**

RR2-10.2196/46995

## Introduction

### Background

The demographic transition to older societies is occurring worldwide [1]. The World Health Organization (WHO) predicts that the number of people aged ≥65 years will double from 703 million in 2019 to 1.5 billion in 2050, rising from 6% to 16% of the planet’s population [1]. The number of people aged ≥80 years is expected to rise from 143 million to 426 million during the same period [1]. Aging exposes people to a variety of health problems and debilitating chronic diseases that can result in functional dependency. Unfortunately, many older adults experiencing multiple chronic conditions of different etiologies can no longer remain at home and must transition to a nursing home (NH) [2]. Depression or other mental disorders also represent a substantial risk of NH admission [3].

Depressive disorders are characterized by depressive mood or loss of pleasure, accompanied by other cognitive, behavioral, or neurovegetative symptoms that persist for at least 2 weeks and significantly affect an individual’s ability to function [4,5]. The *International Classification of Diseases, 11th Edition* defines a depressive episode as having at least 5 out of a list of 10 symptoms, and these must manifest themselves most of the day, nearly every day, or for at least 2 weeks [6,7]. Despite its elevated prevalence of approximately 50%, depression among NH residents is often underdiagnosed and consequently undertreated [8]. With a characteristic pattern of cognitive deficits, mainly affecting executive function, attention, and processing speed, depression affects everyday life [9,10]. NH residents with depressive symptoms often have less ability to perform basic functions, and the condition sometimes speeds them toward functional disability, with an increased risk of falls [11].

Falls are frequent among NH residents, who often already have diminished functional capacities [12]. The WHO defines a fall as “an event in which a person inadvertently lands on the ground or any other surface at a lower level than that at which he was previously standing” [13]. The incidence of falls in NHs is estimated to be twice as high (at 1.7 falls per resident-year) as in the community, and residents are 10 times more likely to sustain a significant injury [14,15]. Indeed, a recent systematic review and meta-analysis estimated the incidence of falls among older adults in NHs to be 43% (95% CI 38-49) [11]. The consequences of falls among NH residents can extend beyond immediate physical harms. Falls can result in serious injuries such as long-standing pain, fractures, and head trauma, which often lead to transfer and admission to hospital, increasing medical costs and impacting the residents’ quality of life [16-18]. Falls increase disability, and injured older adults frequently fail to recover their previous level of function [19]. Hip fractures are associated with high morbidity rates, often leading to a reduced ability to perform daily activities [20-23]. Falls are responsible for a significant number of deaths, making them the second leading cause of death by unintentional or accidental trauma worldwide [24,25]. In addition to being costly for both older adults and the health care system, falls can cause anxiety among health professionals and lead to family complaints [26].

Physical injuries and falls also have psychological consequences [27]. Many people who have experienced a fall are afraid of falling again, leading to immobility, followed by pressure ulcers, pneumonia, weakness, and an increased risk of falls [28]. Excessive fear of falling, which is frequently associated with depression, increases the risk of falls [29]. Both depression and fear of falling are associated with impaired gait and balance, an association that is mediated along cognitive, sensory, and motor pathways. A systematic review and meta-analysis by Gambaro et al [29] highlighted the association between depression and the fear of falling, which in turn increased the likelihood of falls.

Depression has been reported as an independent risk factor for falls and has been shown to increase the risk of future falls [30-32]. Four determinants of recurrent falls are reported: postural sway, history of falls, handgrip strength, and depressive symptoms [33,34]. The relationships between depression, cognitive performance, motor performance, and the risk of falls were also illustrated in a recent study that also found that depression slowed choice stepping reaction time, mediated by quadriceps strength and executive function [35]. Vascular disease and its related burden of white matter lesions may produce concurrent changes in balance, gait, and mood [36]. The interaction between depression and falls may also be self-perpetuating among those who are recurrent fallers, inducing the demoralizing effect of repeated falling.

Although antidepressant medication can mitigate depressive symptoms, which should lower fall risks, it also increases fall risk independent of depression. Recent studies reported that people using antidepressant medication no longer experienced depressive symptoms (with only 18% scoring ≥5 on the 15-item Geriatric Depression Scale [GDS-15]), and the use of antidepressants was an independent risk factor for falls [37,38].

A multifaceted approach is crucial for fall reduction, which includes staff training, systematic use of decision support tools, and implementing falls prevention actions [39]. A recent study demonstrated that a program combining individually prescribed progressive resistance training with balance exercises significantly reduced fall rates and improved physical performance among NH residents [40]. To ensure equitable access and guarantee high-quality care precisely when and where needed, new care models incorporating technological advancements are proving effective and well accepted [41]. Given the evidence that NH residents are particularly susceptible to both depression and falls, this study aims to enhance understanding of these issues within this setting. This is crucial for developing tailored care strategies that address their specific needs effectively.

### Objectives

This integrative review aimed to focus on and synthesize the literature from studies on the relationship between depression and falls among NH residents. The review’s guiding research question was “What is the relationship between depression and falls among NH residents?” The review also sought to briefly report on the factors contributing to falls identified in the retrieved studies.

## Methods

### Design

This study used an integrative review design to synthesize published papers on the relationship between depression or depressive symptomatology and falls among NH residents receiving or not receiving treatment with antidepressant medication. It reported factors contributing significantly to falls based on the guidelines developed by Toronto and Remington [42]. The review rigorously applied the 6 steps of the integrative review process by (1) formulating a review question, (2) systematically searching for and selecting literature, (3) assessing the quality of the studies selected, (4) analyzing and synthesizing studies retained, (5) discussing new knowledge, and (6) proposing further steps for a dissemination plan [43]. Regarding the protocol for this integrative review, published in *JMIR Research Protocols* (ISSN 1929-0748) [44], the authors modified the exclusion criteria for article types and the statistical analysis. In line with the concepts of an integrative review, which impose no restrictions on the designs of the studies evaluated, we considered some study designs other than primary studies, including literature reviews. In addition, no meta-analysis of the results on the relationships between depressive symptoms and depression and falls was computed.

### Inclusion and Exclusion Criteria

This review considered papers reporting on falls and their relationship with depression or depressive symptoms among older adults with a mean or median age of ≥65 years living long term in NHs. NHs were considered institutions providing 24-hour room and board services and assistance with the activities of daily living (ADLs) or the instrumental ADLs, as well as health services for the management of the chronic conditions of older adults with physical and cognitive or mental impairments [45]. Textbox 1 presents the complete inclusion and exclusion criteria.

**Inclusion and exclusion criteria.**

**Inclusion criteria**

- Population: older adults aged ≥65 years
- Depression: depression (diagnosed by a medical professional) or depressive symptoms (assessed by health care professionals using validated tools)
- Falls: incidence of falls, prevalence of falls, and reported or assessed risk of falls
- Health care setting: nursing homes and geriatric or psychiatric nursing homes
- Article types: original prospective research studies with a descriptive, correlational, or cohort design; retrospective cohort studies; mixed methods studies; and literature reviews
- Language: no restrictions

**Exclusion criteria**

- Population: adults below the age of 65 years
- Depression: antidepressant medication prescription without diagnosed depression or recognized depressive symptoms
- Falls: near falls
- Health care setting: intrahospital units, older adults’ houses or apartments, community care living, and assisted living apartments
- Article types: meeting abstracts, conference abstracts, posters, guidelines, commentaries, editorials, opinion papers, book reviews, and case reports

### Information Sources and Search Strategy

A literature search of the following bibliographic databases was conducted in October 2023 in collaboration with a medical librarian (JRA): MEDLINE ALL Ovid, Embase, CINAHL with Full Text EBSCO, APA PsycInfo Ovid, Web of Science Core Collection, the Cochrane Database of Systematic Reviews Wiley, and ProQuest Dissertations & Theses A&I. Clinical trials were searched for in the Cochrane Central Register of Controlled Trials Wiley, ClinicalTrials.gov, and the WHO’s International Clinical Trials Registry Platform. Searches were performed without language or date restrictions. Additional searches were performed using Google Scholar, the DART-Europe E-theses Portal, and backward citation tracking. Multimedia Appendix 1 provides details of the search syntax, keywords, and index terms used.

### Selection Process

The initial search yielded 2345 references, and after the removal of duplicates, 1337 unique citations were identified and imported into EndNote (Clarivate) software. Two reviewers (AMQ and HV) independently screened the titles and abstracts to identify documents to be included in the second phase. Disagreements over whether studies met the inclusion and exclusion criteria were resolved by discussion between the reviewers. A total of 105 studies were identified as suitable for a full-text review and were independently assessed by 2 reviewers (AMQ and HV).

Backward citation searching identified 6 further studies, but they failed to meet the inclusion criteria. The coauthors discussed and resolved disagreements over inclusion (AMQ, AvG, MMM, AML, and HV). Finally, 7 studies were identified as thoroughly matching the inclusion and exclusion criteria. These studies then underwent a quality appraisal and, finally, continued to the data extraction step. The 2020 PRISMA (Preferred Reporting Items for Systematic Reviews and Meta-Analyses) standards were followed for reporting information from each step of the review selection process [46].

### Data Extraction

Two reviewers (AMQ and HV) developed the data extraction process using a specific, structured data extraction matrix. The following variables were included to answer the research question and meet the integrative review’s aims: (1) authors, year of publication, country location, and study duration; (2) study design and sample size; (3) participants’ characteristics, including sex, mean or median age, and SD or interquartile 25th to 75th percentile; (4) information about depression or depressive symptoms, depression evaluation tools, information about falls; (5) statistical results about the relationship between depression and falls; and (6) factors contributing to falls.

### Assessment of the Risk of Bias in Included Studies

The quality of studies retained was assessed using the Newcastle-Ottawa Scale (NOS) [47] and the Appraisal tool for Cross-Sectional Studies (AXIS) [48]. The NOS comprises 2 different scales designed to evaluate cohort or case-control studies [47]. The NOS scale enables the attribution of scores, up to a total maximum score of 9, in 3 different domains: selection (maximum score 4), comparability (maximum score 2), and outcome (maximum score 3). The AXIS was developed by a panel of experts using a Delphi methodology, and it focuses principally on studies’ published methods and results [48]. Two authors (AMQ and HV) performed the evaluations independently. Each study’s 3 evaluation domains were meticulously analyzed to evaluate each domain and item based on the reported information. Any assessment score disagreements were resolved through discussion until a consensus was reached, and no third author had to be solicited.

### Statistical Analyses

Descriptive statistics of the population’s median age, the distribution of men and women, and the number of falls recorded in the studies retained were computed. Additional descriptive statistics on the prevalence of falls and depressive symptoms were computed to report differences between NH residents’ profiles and types of NH.

In line with integrative review methodology, which focuses on the comprehensive analysis and synthesis of research findings, a meta-analysis was not conducted, given the substantial heterogeneity among the retained studies [42,49]. Data were analyzed using SPSS software (version 29.0; IBM Corp).

## Results

### Overview

**Figure 1:**
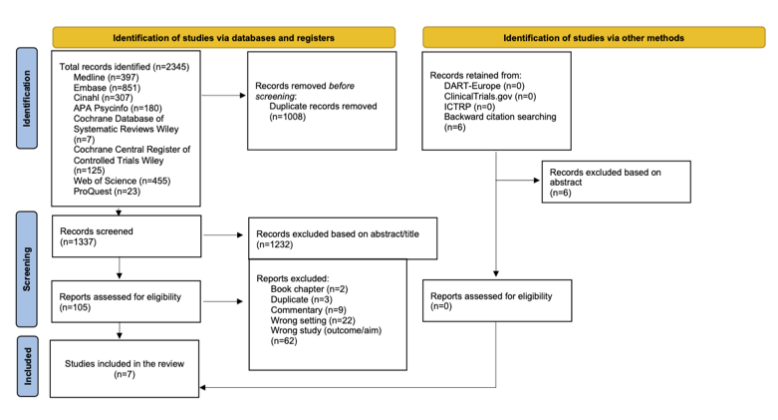
The integrative review flow diagram. ICTRP: International Clinical Trials Registry Platform.

The selection process (Figure 1 [46]) identified 7 eligible articles that rigorously met the review’s inclusion criteria, including a description of the study population, measures of depressive symptoms, fall measures, and statistical results for the relationship between depression or depressive symptoms and falls, summarized in Tables 1 and 2.

**Table 1:** Characteristics of the studies retained.

Study	Country	Study aims	Design, data, and period	Sample size, N	Sex	Age (y)	Scales of depression assessment
Kioh and Rashid [50], 2018	Malaysia	Determine the prevalence and risk of falls and their associated factors among nursing home residents	Cross-sectional study collected data from October 2016 to July 2017 in 10 nursing homes	354	Female=230 (65%); male=124 (35%)	Age groups: 60-69, n=47 (13.3%); 70-79: n=162 (45.7%); 80-89, n=145 (41.0%)	GDS-15^a^
Damian et al [51], 2013	Spain	Measure the frequency of falls and associated factors among older adults living institutions	Cross-sectional study collected data from June 1998 to June 1999 in 55 living institutions	733	Female=408 (76%); male=325 (24%)	Mean 83.4 (SD 11.5% ; 95% CI 82.6-84.1)	GDS-10^b^
Khater and Mousa [52], 2012	Egypt	Evaluate the incidence of falls and the risk factors among nursing home residents	Prospective cohort study collected data from June 2009 to May 2010 in 3 nursing homes	84	Female=48 (57.1%); male=36 (42.9%)	Mean 71.9 (SD 7.2)	GDS-15
Ku et al [53], 2013	Taiwan	Investigate the prevalence and frequency of falls and identify factors of falls among older adult men in Taiwan	Cross-sectional study collected data from December 2009 to December 2010 in 4 long-term stay veterans’ settings	940	Male=940 (100%)	Mean 85.5 (SD 5.72)	GDS-15
Wang et al [54], 2012	China	Examine the combined effects of medical conditions and depression status on fall incidents among institutionalized older adults	Cross-sectional study collected data over 1 year in 4 long-term care institutions	286	Female=193 (67.5%); male=93 (32.5%)	Mean 81.9 (SD 6.16)	GDS-15
Sylliaas et al [55], 2012	Norway	Examine whether the severity of dementia, behavioral and psychological symptoms, and depression can predict falls among nursing home residents	Cohort study with a 1-year follow-up collected data from November 2004 to January 2025 in 20 nursing homes	1147	Female=839 (73.1%); male=308 (26.9%)	Mean 84.8 (SD 6.86)	Cornell Scale for Depression in Dementia
Kron et al [56], 2003	Germany	Identify individual predisposing risk indicators for falls in a sample of institutionalized, frail older adults	Prospective observational study collected data from October 1998 to September 1999 in 3 long-term care institutions	472	Female=363 (77%); male=109 (33%)	Mean 84.0 (SD 7.0)	Minimum Data Set of the Resident Assessment Instrument, version 2.0.

^a^GDS-15: 15-item Geriatric Depression Scale.

^b^GDS-10: 10-item Geriatric Depression Scale.

**Table 2:** Depressive symptomatology and falls or risk of falls among nursing home residents.

Study	Statistical results of depression and depressive symptoms	Statistical results of falls	Statistical results for the relationship between depression or depressive symptoms and falls or risk of falls	Other findings on the relationship with falls or risk of falls	Main findings about the relationship between depression or depression symptoms and falls
Kioh and Rashid [50], 2018	Depression (GDS^a^ score) n=76 (21.5%)	Fallers: n=116 (32.8%); 1 fall: n=68 (19.2%); >1 fall: n=48 (13.6%); Risk of fall (Fall Risk Assessment Tool): low risk: n=307 (86.7%); moderate-high risk: n=47 (13.3%)	Depression and falls: OR^b^ 1.71, 95% CI 1.00-2.91; P<.05 Depression and risk of falls: OR 1.83, 95% CI 1.06-4.23; P<.05	Respiratory illnesses and falls: OR 3.38, 95% CI 1.11-10.30; P<.05 History of >1 fall in the past 12 months and risk of falls: OR 3.90, 95% CI 1.72-8.8; P<.05	Depression was strongly associated with the prevalence of falls and the risk of falls. Respiratory illness was also significantly associated with falls. A history of >1 fall in the past 12 months was significantly associated with the risk of falls.
Damian et al [51], 2013	Depression (disease) n=154 (21%) Depressive symptoms (GDS score) n=193 (33%)	Fallers: n=88 (12%; 95% CI 9-15), 1.5 falls/person-year Falls: n=146, 2.4 falls/resident-year (95% CI 2.04-2.82); 1 fall: n=60 (68%); 2 falls: n=18 (21%); ≥3 falls: n=10 (12%) 1-year risk of falling=0.78	Depression and falls: RR^c^ 2.49, 95% CI 1.38-4.50; RR 1.55, 95% CI 0.95-2.5 Depressive symptoms (GDS score) and falls: RR 1.06, 95% CI 0.95-1.19; RR 1.01, 95% CI 0.92-1.1	Number of diseases ≥1: RR 1.40, 95% CI 1.27-1.54; RR 1.32, 95% CI 1.17-1.50 Urinary incontinence: RR 2.89, 95% CI 1.48-5.65; RR 2.56, 95% CI 1.32-4.94 Arrhythmias: RR 3.36, 95% CI 1.80-6.30; RR 2.00, 95% CI 1.05-3.81 Antidepressants: RR 3.40, 95% CI 1.65-7.04; RR 2.32, 95% CI 1.22-4.40	Depression was associated with falls when adjusted for age, sex, cognitive status, and functional dependence. Multimorbidity, urinary incontinence, arrhythmias, and antidepressants were the most relevant factors for falls.
Khater and Mousa [52], 2012	Depression (GDS score): n=39 (46.4%) GDS score: mean 4.61 (SD 2.55)	Fallers: n=53 (63.1%) Falls: n=163, 5.3 falls/1000 resident days; 631 fallers/1000 resident-years; 1940 falls/1000 resident-years)	GDS Fallers: mean 4.8 (SD 2.5); P=.51 Nonfallers: mean 4.4 (SD 2.7); P=.51	Frailty: OR 2.340, 95% CI 1.542-16.746; P=.01 Timed Up and Go test: OR 3.271, 95% CI 1.287-19.539; P<.001	Depression did not differ significantly between fallers and nonfallers. Frailty and poorer results in Timed Up and Go tests were the only independent risk factors for falls.
Ku et al [53], 2013	Depression (GDS score) n=334 (35.5%); GDS: mean 4.3 (SD 4.05)	Fallers: n=162 (17.2%); 1 fall: n=97 (59.9%); 2 falls: n=31 (19.1%); 3 falls: n=17 (10.5%); 4 falls: n=7 (4.3%); 5 falls: n=6 (3.7%); 6 falls: n=1 (0.6%); ≥7 falls: n=3 (1.9%) Fall incidence: mean 3.6 (SD 2.5)	Depression and falls: OR 1.05, 95% CI 1.01-1.10; P=.01	Advanced age: OR 1.04, 95% CI 1.01-1.07; P=.02 Stroke: OR 2.16, 95% CI 1.18-3.96; P=.01 Gout: OR 1.96, 95% CI 1.36-2.81; P<.001 Cataract: OR 1.48, 95% CI 1.03-2.15; P=.04	Depression was an independent variable for predicting falls, as were increasing age, stroke, gout, and cataracts.
Wang et al [54], 2012	Depression (GDS score) n=68 (23.8%)	Fallers: n=81 (28.3%)	Depression and risk of fall: OR 1.92, 95% CI 1.00-3.64; P<.05	Ancillary device use and risk of falls: OR 2.70, 95% CI 1.50-4.86; P<.01 Medications ≥4 and risk of falls: OR 3.23, 95% CI 1.44-7.26; P<.01 Depression, ancillary device, and risk of fall: OR 6.52, 95% CI 2.62-16.3; P<.01 Depression, neural system diseases and risk of fall: OR 11.3, 95% CI 1.96-65.2; P<.01 Depression, medication ≥4 and risk of fall: OR 5.28, 95% CI 1.46-19.1; P<.05	Depression status, using ancillary devices, and multiple medication use were significantly associated with the risk of falling.
Sylliaas et al [55], 2012	Cornell Scale for Depression in Dementia: mean 5.35 (SD 5.1)	Fallers: n=459 (40%); 1 fall: n=459 (40.0%); ≥2 falls: n=379 (33.0%)	CSDD^d^ and falls: RR 1.37, 95% CI 1.13-1.65; P=.001	NPI^e^: RR 1.15, 95% CI 1.08-1.19; P<.001 Age: RR 1.03, 95% CI 1.01-1.04; P=.002 CDR^f^ Scale: RR 1.06, 95% CI 1.03-1.08; P<.001 PADL^g^: RR 0.95, 95% CI 0.92-0.97; P<.001 Use of sedatives: RR 1.08, 95% CI 1.04-1.23; P=.003	Severe depression (higher scores on CSDD) significantly predicted ≥1 falls in a bivariate Cox regression. Age; higher scores on the NPI, CDR, and PADL; and use of sedatives were all independent predictors in a multivariate regression analysis.
Kron et al [56], 2003	Minimum Data Set of the Resident Assessment Instrument, version 2.0: n=225 (47.7%)	Fallers: n=247 (52.3%), 645 fallers/1000 resident-years, 2.558 falls/1000 resident-years Frequent fallers: n=115 (24.4%)	Depression and >2 falls: OR 1.6, 95% CI 1.0-2.6; P=.049	Total transfer assistance: OR 0.4, 95% CI 0.2-0.7; P=.002 Urinary incontinence: OR 2.1, 95% CI 1.2-3.6; P=.007 Fall in last 180 days: OR 5.2, 95% CI 3.2-8.5; P<.001	Depression, transfer assistance, urinary incontinence, and a positive fall history were important risk indicators associated with the risk of ≥2 falls.

^a^GDS: Geriatric Depression Scale.

^b^OR: odds ratio.

^c^RR: risk ratio.

^d^CSDD: Cornell Scale for Depression in Dementia.

^e^NPI: Neuropsychiatric Inventory.

^f^CDR: Clinical Dementia Rating.

^g^PADL: Personal Activity of Daily Living.

### Characteristics of the Included Studies

Most of the studies were published between 2012 and 2018 [50-55], with one published in 2003 [56]. The papers were written by 7 different first authors and examined 7 countries (Malaysia, Spain, Egypt, Taiwan, China, Norway, and Germany), with 43% (3/7) of the studies carried out in Europe [51,55,56], 43% (3/7) in Asia [50,53,54], and 1 (14%) in Africa [52]. Of the 7 studies, 6 (86%) were cross-sectional [50,51,53-56], and 1 (14%) was a prospective cohort study [52].

### Population

The studies included a total of 4016 participants, with a mean of 574 (SD 380) older adult participants and a significant variance in numbers from 84 [52] to 1147 participants [55]. The total sample contained 48.2% (1935/4016) men and 51.8% (2081/4016) women. The median age of the overall sample was 83 (IQR 79.4-84.9) years, with minimum and maximum ages of 71.9 and 85.5, respectively.

### Long-Term-Care Facility Definitions

The review considered studies focusing on NH residents with at least a mean or median age of 65 years and living in a geriatric or psychiatric NH, although different terms were used. Ku et al [53] examined 4 long-term stay settings for male veterans only. Of the 7 studies, 3 (43%) [50,52,55] specifically mentioned NHs as their setting, 2 (29%) referred to long-term care institutions [54,56], 1 (14%) described its location as a long-term stay setting [52], and 1 (14%) described them as older people living institutions [51].

### Aims of the Studies

The 7 studies retained examined relationships between depression or depressive symptoms and falls in different ways. One cross-sectional study [54] focused directly on the directional relationship between depression status and falls. Another cross-sectional study [55] examined the effects of the severity of dementia, behavioral and psychological symptoms, and depression on falls. The studies retained frequently aimed to describe the risk factors for and factors associated with falls, including 4 cross-sectional studies [50,51,53,56] and 1 cohort study [49].

### Depression

#### Detection and Diagnosis of Depression

Several different depressive symptom measurement tools were used. The 15-item and 10-item Geriatric Depression Scales (GDS) were most commonly used, with 57% (4/7) of the studies [50,52-54] using the GDS-15 and 14% (1/7) of the studies using the GDS-10 [51]. Of these 4 studies, 2 (50%) [53,54] defined depression as a GDS score≥5, but 1 (25%) study [50] categorized participants with a score of 0 to 5 as normal. The study using the GDS-10 [51] scored 0 to 3 as normal and scores of 4 to 7 and 8 to 10 as moderate and severe depressive symptoms, respectively. One study using the GDS-15 [52] did not describe its categorization of depressive symptoms. One study used the Cornell Scale for Depression in Dementia to measure depressive symptoms [55], and the final study used indicators of the Minimum Data Set of the Resident Assessment Instrument, version 2.0 [56]. Neither of the studies mentioned how they categorized depressive symptoms. Two studies specified having based their depression assessments on interviews with residents about their status over the previous 7 days [51] and self-related depressive symptoms [54]. One study [55] was based on information from NH staff members. The other 4 studies [51-53,56] did not specify this information. Only one of the studies revealed a medical diagnosis of depression [51]. Multimedia Appendix 2 [50-56] presents the depression measurement strategies used by the studies retained.

#### Prevalence of Depression

Of the 7 studies, 6 (86%) included [50-54,56] showed a prevalence of depression above 20%. The mean prevalence of depressive symptoms in Asian studies was 26.9%, with a significant variation observed between countries [50,53,54]. Ku et al [53] and Wang et al [54] used the GDS-15 scale, with identical cutoff scores, resulting in an overall mean prevalence of depression of 32.8% (402 participants with depressive symptoms/1226 participants). The lowest prevalence of depression was 21.5% (76 participants with depressive symptoms/354 participants) as reported by Kioh and Rashid [50]. In the European studies, Kron et al [56] reported the highest prevalence of depression, at 47.7%, using data from the MDS-RAI 2.0 assessments. Damian et al [51] reported a 33% prevalence of depressive symptoms using the GDS-10 scale. The study by Sylliaas et al [55] reported unclear data on the prevalence and limited the data collected to reporting the odds ratio (OR) of the relationship between depression and falls. No additional data was available. The study by Khater and Mousa [52], performed in Egypt, revealed a notably high prevalence of depression, at 46.4%.

### Falls

#### Descriptions of Falls

The studies included described falls heterogeneously. Khater and Mousa [52] and Wang et al [54] referred to the studies by Delbaere et al [57,58] to define a fall as an unexpected event in which the person comes to rest on the ground, the floor, or a lower level. Kioh and Rashid [50] and Kron et al [56] described falls as unintentionally coming to rest on the ground, the floor or another lower level, whether accidentally or nonaccidentally, other than as a consequence of the sudden onset of paralysis, an epileptic seizure or an overwhelming external force. Damian et al [51] did not define falls. Ku et al [53] based their definition on Tinetti’s, which defines a fall as an event that results in a person coming to rest unintentionally on the ground or another lower level, not due to any intentional movement, a major intrinsic event (eg, stroke) or an extrinsic force (eg, being forcefully pushed down, knocked down by a car) [59]. Finally, Sylliaas et al [55] referred to Lord [60], who defined a fall as an unexpected event in which the participant rests on the ground, the floor or at a lower level.

#### Frequency of Falls or Fall Risks

Different methods were used to collect fall frequency. Two studies [50,54] collected data on the history of falls in the past 12 months through a resident questionnaire and face-to-face interviews. Kioh and Rashid [50] also used the Fall Risk Assessment Tool to determine fall risk. Damian et al [51] used physician and nurse interviews and annotations to determine the number of falls in the preceding 30 days and used the Timed Up and Go test to assess fall risk. Ku et al [53], Sylliaas et al [55], Khater and Mousa [52], and Kron et al [56] explored data collected on events recorded during the study period. Wang et al [54] considered participants who had had a single fall or no falls as “nonfallers,” and all other participants with ≥2 falls or with at least 1 injurious fall as “fallers.” Kron et al [56] distinguished “fallers” (participants with 1 or 2 falls) from “recurrent fallers” (participants with >2 falls). Multimedia Appendix 3 [50-56] presents the retained studies’ definitions and measurement strategies for falls.

#### Prevalence of Falls

All the studies reported the number of residents who had at least one fall during the study period. Of the total combined sample of 4016 participants, based on each study’s definition or description of a fall, 1206 (30%) fell at least once. The highest prevalence of fallers was 63%, with 53 of a sample of 84 participants falling during the 1-year follow-up period in the study by Khater and Mousa [52] in Egypt. The mean prevalence of falls in the studies conducted in European countries was 34.8% (SD 16.86%). The lowest prevalence was 12%, with 88 of 733 participants having at least one fall in the preceding 30 days in the study by Damian et al [51]. Fallers made up 52.3% (247 fallers/472 participants) in the study by Kron et al [56] and 40% (459 fallers/11,147 NH participants) in the study by Sylliaas et al [55]. The prevalence of fallers in Asian studies showed a mean of 26.1% (SD 5.6%). During the year of their study, 162 (17.2%) of 940 participants fell in the study by Ku et al [53], 81 (28.3%) of 286 participants fell in the study by Wang et al [54], and 116 (32.8%) of 354 participants fell in the study by Kioh and Rashid [50]. Ku et al [53] reported a single-fall prevalence of 10.3% (97 fell once/940 male veteran participants) and a recurrent-falls prevalence of 6.9% (65 with a history of falls fell/940 participants). Three studies [51-53,56] reported fall incidence rates. In the study by Khater and Mousa [52], 163 falls occurred among 53 fallers, equivalent to 1940 falls/1000 resident-years (5.3 falls/1000 resident-days). Kron et al [56] recorded 980 falls (2.558 falls/1000 resident-years) among 247 fallers (645 fallers/1000 resident-years). With a total number of 146, Damian et al [51] reported an incidence of 2.4 falls (95% CI 2.04-2.82) per resident-year. On the basis of the FRAT scores, Kioh and Rashid [50] identified 307 (86.7%) participants at a low risk of falls and 47 (13.3%) participants at a moderate or high risk of falls.

### The Relationship Between Depression and Falls

The main retrieved publications in our integrative review used the OR to examine the relationship between depression and falls among NHs residents [50,52-54,56]. The OR was used to quantify the association between exposure to depressive symptoms and the occurrence of falls. Consequently, the findings of these studies reflected the odds of falls occurring in the presence of depressive symptoms.

The remaining studies used the risk ratio (RR) to study the mentioned relationship [51,55]. The RR allowed for a comparison of the probability of falls occurring in NH residents with depressive symptoms versus those without depressive symptoms.

Four studies [50,53,54,56] identified a significant association between depression and falls among NH residents. Kioh and Rashid [50] revealed that having depression (OR 1.80, 95% CI 1.07-3.04) was a significant factor associated with falls. After performing a binary logistics regression to take account of possible confounders, depression was also associated with the prevalence of falls among participants (OR 1.71, 95% CI 1.00-2.91) [50]. Ku et al [53] showed that fallers tended to have more severe depression than nonfallers (44.4% vs 33.7%, respectively; *P*=.01). Using multivariate logistic regression analyses, they revealed that depression status (OR 1.05, 95% CI 1.01-1.10; *P*=.01) was an independent variable for predicting falls [53]. On the basis of the univariate analysis, Wang et al [54] found depression to be significantly associated (OR 2.00, 95% CI 1.13-3.55; *P*<.05) with the risk of falling. In addition, they reported that depression also had a significant association in a multivariate logistic regression analysis (OR 1.92, 95% CI 1.00-3.64; *P*<.01). Kron et al [56] revealed that depressive symptoms were a significant risk indicator (OR 1.6, 95% CI 1.0-2.6; *P*=.049) for predicting recurrent falls among their participants. Damian et al [51] reported an association between depression and falls (RR 2.49, 95% CI 1.38-4.50); however, they found a mediating effect of antidepressant and anxiolytic medication (RR 1.55, 95% CI 0.95-2.51). Khater and Mousa [52] showed that levels of depression were not significantly different between fallers and nonfallers. Sylliaas et al [55] found that severe depression was 1 of the 4 strongest predictors of falls (RR 1.38, 95% CI 1.13-1.65; *P*=.001), but it was no long a significant independent variable after multivariate regression analysis (RR 0.99, 95% CI 0.96-1.02; *P*=.55).

A total of 19 potential risk factors, other than depression, were associated with falls among NH participants. Kioh and Rashid [50] showed a significant association between respiratory illnesses and falls (OR 3.38, 95% CI 1.11-10.30; *P*<.05) as well as between a history of >1 fall in the past 12 months and risk of falls (OR 3.90, 95% CI 1.72-8.8; *P*<.05). A history of falls in the last 180 days was also identified as an important fall risk indicator (OR 5.2, 95% CI 3.2-8.5; *P*<.001) in the study by Kron et al [56]. Kron et al [56] documented that urinary incontinence was one of the most relevant factors for falls (OR 2.1, 95% CI 1.2-3.6; *P*=.007), and in the study by Damian et al [51], it was an important risk indicator associated with ≥2 falls (RR 2.89, 95% CI 1.48-5.65). Wang et al [54] found that polypharmacy was significantly associated with the risk of falls (OR 3.23, 95% CI 1.44-7.26; *P*<.01). Damian et al [51] found that antidepressants were a relevant factor for falls (RR 3.40, 95% CI 1.65-7.04), and Sylliaas et al [55] reported sedatives as an independent predictor of an increased risk of falling (RR 1.08, 95% CI 1.04-1.23; *P*=.003). In addition, Damian et al [51] found that multimorbidity (RR 1.40, 95% CI 1.27-1.54) and arrhythmias (RR 3.36, 95% CI 1.80-6.30) were relevant risk factors for falls. Stroke (OR 2.16, 95% CI 1.18-3.96; *P*=.01), gout (OR 1.96, 95% CI 1.36-2.81; *P*<.001), cataracts (OR 1.48, 95% CI 1.03-2.15; *P*=.04), and advanced age (OR 1.04, 95% CI 1.01-1.07; *P*=.02) were independent variables for predicting falls in the study by Ku et al [53]. Age was also identified as an independent predictor of a risk of falls in the study by Sylliaas et al [55].

Frailty (OR 2.340, 95% CI 1.542-16.746; *P*=.01) and poorer results in Timed Up and Go tests (OR 3.271, 95% CI 1.287-19.539; *P*<.001) were the only independent risk factors for falls in one study [52]. Wang et al [54] found that ancillary device use increased the risk of falls (OR 2.70, 95% CI 1.50-4.86; *P*<.01) and that the risk of falling was enhanced by the interacting factors of depression, ancillary devices (OR 6.52, 95% CI 2.62-16.3; *P*<.01), nervous system diseases (OR 11.3, 95% CI 1.96-65.2; *P*<.01), and >4 medications (OR 5.28, 95% CI 1.46-19.1; *P*<.05). Finally, in one study, higher scores on the Neuropsychiatric Inventory, Clinical Dementia Rating scale, and Personal Activity of Daily Living were all independent of other predictors of an increased risk of falling [55].

### Assessment of the Risks of Bias in the Studies Retained

The quality and risks of bias of the studies retained were assessed using the NOS [47] and AXIS [48]. One cohort study [52] was classified as being of good quality, scoring 8 stars, with 3 in the selection domain, 2 in the comparability domain, and 2 in the outcome domain. Of the 6 cross-sectional studies [50,51,53-56], 4 (67%) [50,51,53,54] were considered of good quality and 2 (33%) of moderate quality [55,56] but sufficient to be integrated into our integrative review and undergo data extraction. Multimedia Appendix [52] 4 and 5 [50-56] summarize the quality evaluation results of the cross-sectional studies and the quality and risk of bias assessments of the cohort study.

## Discussion

### Principal Findings

This integrative review rigorously searched for publications on the relationship between depression or depressive symptoms and falls among NH residents. A total of 7 studies were selected, with 6 (86%) published in the last 10 years and 1 (14%) published in 2003. The studies included 4016 participants with an overall median age of 83 years. Of the 7 studies, 6 (86%) reported a prevalence of depression >20%, and approximately 30% of the total sample had experienced at least one fall. Despite the methodological differences between the studies, their results predominantly showed a significant relationship between depressive symptoms and depression and falls and the risk of falls [50,51,53-56]. To the best of our knowledge, this was the first review to synthesize relevant publications on how depression is related to falls among NH residents. The results highlight the significance of falls and depressive symptoms among NH residents.

Most of the studies had small sample sizes (Table 1), but most of them had a sufficient sample size individually, and they gave the total sample the necessary statistical power [50-54]. The studies did not all use the same precise definition of an NH or an NH resident. A fall was defined in different ways: some studies used the variable of risk of falls or near falls, and one study considered a resident who had only had one fall to be a nonfaller, making a detailed comparison between fall prevalence between studies somewhat of a conundrum. These differences can be explained by the studies’ designs and geographical contexts and the use of heterogeneous fall assessment tools. To increase comparability and improve the meta-analysis of existing data, it is advisable that future studies adopt a universal description of a fall, for example, the WHO’s definition [61]. Nonetheless, as previously mentioned, the heterogeneous clinical practices of different countries make comparison difficult. In addition, because some of the studies retained only received a moderate quality assessment, any interpretations should be made with care. The review revealed that the relationship between depression and falls is complex and multifactorial, and that the mechanisms of this relationship were not always completely understood [62]. All the studies considered other risk factors. The studies retained were unclear about the methods used to diagnose depression, and although these were primarily based on validated tools, they failed to mention whether physicians had confirmed the diagnoses. The average occurrences of depression reported in the studies retrieved were similar to other studies conducted in NHs [63,64]. Our studies used 4 different validated tools for depression, and there was not always consistency in the cutoff scores used with the same diagnostic tools. In the study by Kioh and Rashid [50], participants with GDS-15 scores of 0 to 5 were categorized as normal, which may explain its lowest prevalence of depression (76 depressed residents/354 participants). This corroborated the findings from similar studies conducted among community-dwelling older adults [65,66]. The literature suggests that many NH residents could have unidentified and thus untreated depression [67]. This could be explained by the potential stigma surrounding mental illness or the idea that depression is a normal state among NH residents and thus has no additional consequences on their health or social life.

Our integrative review did not include publications that took into account NH residents receiving antidepressant medication without a diagnosis of depression or without reported depressive symptoms. Although it was difficult to discriminate whether it was low mood or medication use that predisposed older adults to falls. Kvelde et al [68] found that depressive symptoms remained a risk factor for falls in a large subgroup of older adults not taking antidepressant medications.

Our results seem to support the hypothesis of an interdependent association between the presence of depression and falls or a risk of falls, despite the high percentage of cross-sectional studies preventing any inference of the association’s direction [50,51,53-56]. This is consistent with the clinical review by Iaboni et al [35], which stated that depression and falls had a significant bidirectional relationship. An excessive fear of falling, which is frequently associated with depression, also increases the risk of falls. Both depression and the fear of falling are associated with impaired gait and balance, an association that is mediated through cognitive, sensory, and motor pathways, and which consequently allows us to concur on the bidirectional relationship between depression and falls [35].

In addition, NH residents form a huge population of older adults with a significant number of comorbidities that can contribute to falls. Using different strategies to assess the risk of falls [65] makes causality and relationships extremely difficult to demonstrate. In the studies addressing NH residents that we retained, the prevalence of falls was 30% (1206 fallers/4016 participants), varying over the years. The risk factors of fall history, impaired performance in the ADLs, and insomnia all had strong associations with all falls. Risk factors with low to moderate ORs were vertigo; walking aids; poor balance; use of antidepressants, benzodiazepine, antipsychotics, and anxiolytics; polypharmacy; dementia; unsteady gait; hearing problems; and being male. Having bed rails was identified as a protective environmental factor.

Considering the limitations of this integrative review, current knowledge and the call to action laid out by Matos Queiros et al [44], the bidirectional relationship between depression and falls among NH residents has been inadequately studied. There is an urgent need to clarify their complex interplay [35], and this will require robust, high-quality research. Our literature search strategy found many references (Figure 1 [46]); however, surprisingly, few studies considered the relationship between depression and falls among NH residents as one of their principal variables of interest—depression was regarded more as a contributing factor [16,29]. Most studies focused on the relationships between neurodegenerative diseases, functional impairment, and falls, as well as the associations between these factors and medication prescriptions for NH residents [69,70]. Another concern was that most of the studies were intervention studies to prevent or reduce falls and fall risks among NH residents [71,72]. Another avenue of research involved falls among community-dwelling older adults in assisted living facilities or their own homes [73-76].

Depression may lead to an increased risk of falls through behavioral, neuromuscular, or pathological pathways, and coherent with the fact that depressed NH residents are mostly less active than those without depression [77]. Moreover, the lower sense of self-efficacy and negative expectations about the future found among repeated fallers with depression can lead to decreased social participation [78].

Furthermore, our integrative review’s findings may draw attention to the need to enhance comprehensive geriatric assessment practices as a suitable, more multidimensional, multidisciplinary approach to addressing NH residents’ complex needs [79]. Specifically, health care professionals should implement regular screening for depressive symptoms as part of comprehensive fall risk assessments [79]. This proactive approach could lead to the early introduction of targeted therapeutic strategies to address depressive symptoms among NH residents, and it could also yield the positive outcome of reducing fall incidents [68]. In addition, fostering interdisciplinary collaboration among health care professionals, including old-age psychiatrists, nurses, and physiotherapists, should be encouraged to create a holistic, person-centered care approach [80,81].

### Study Limitations

This integrative review had some limitations. Despite a thorough literature search using recognized methodological guidelines and recommendations, the review may have missed some studies meeting all the selection criteria due to search errors or investigators’ mistakes. The studies retrieved evaluated NH residents’ clinical state of depression using self-reported data, which could have created a risk of bias. In addition, studies did not clearly specify if there was a physician diagnosis of depression. The studies selected mainly used depression as an independent variable of interest, which may have led to this variable’s overrepresentation compared to other factors affecting falls. Substantial heterogeneity in the classification and measurement of falls and depressive symptoms resulted in studies using different constructs, making comparisons difficult. Finally, any generalization of the present findings should be made cautiously, as the NHs and residents studied were always from a particular region or country.

### Conclusions

By synthesizing existing research and drawing conclusions from diverse studies, this integrative review provides a more holistic understanding of the relationship between depression and depressive symptoms and falls. Our findings suggested that depression was a potential and independent risk factor for falls among NH residents. Moreover, depression and depressive symptoms should be addressed and systematically evaluated so that nursing professionals can implement preventive measures. Our results underscore the importance of policies that invest in and support the implementation of depression and fall risk assessment guidelines within NHs. These are essential for developing personalized care plans, thereby reducing fall risks and enhancing NH residents’ overall quality of care and life. Although the precise associations of depression and depressive symptoms have yet to be clarified, it seems certain that they are risk factors for falls. The small number of studies published on our phenomenon of interest emphasizes the need for further research. Further longitudinal studies are urgently needed to elucidate the complex relationship between depression and falls in NHs. Considering their serious consequences, depression and falls among NH residents are a significant public health problem and require urgent attention from frontline health care professionals, researchers, and policy makers. Addressing these gaps through rigorous research will be critical to informing effective interventions and enhancing outcomes for NH residents.

## Appendix

Multimedia Appendix 1 Search strategies.

Multimedia Appendix 2 Detection and diagnosis of depression.

Multimedia Appendix 3 Definition and measurement of falls.

Multimedia Appendix 4 Methodological quality of the retained cross-sectional studies according to the Appraisal tool for Cross-Sectional Studies (YN score).

Multimedia Appendix 5 Methodological quality of the cohort study retained based on the Newcastle–Ottawa Scale guidelines.

## Abbreviations

ADL: activity of daily living
AXIS: Appraisal tool for Cross-Sectional Studies
GDS-15: 15-item Geriatric Depression Scale
NH: nursing home
NOS: Newcastle-Ottawa Scale
OR: odds ratio
PRISMA: Preferred Reporting Items for Systematic Reviews and Meta-Analyses
RR: risk ratio
WHO: World Health Organization

## Data Availability

The datasets used in this study are available from the corresponding author upon reasonable request.
