# Supplementary material for: Relationship Between Depression and Falls Among Nursing Home Residents: Integrative Review
Source: Interact J Med Res. 2024 Nov 28;13:e57050. doi: 10.2196/57050 (PMC11638692; doi:10.2196/57050)
Supplement: Multimedia Appendix 1 [file ijmr_v13i1e57050_app1.docx]

Multimedia Appendix 1.

Search strategies

Joëlle Rosselet Amoussou, Medical Library-Cery, Lausanne University Hospital and University of Lausanne, Site de Cery, 1008 Prilly, Switzerland, ORCID 0000-0001-6871-5350

The research strategies were peer reviewed by another information specialist prior to execution.

**Embase.com**

851 references found, 27 October 2023

('nursing home'/exp OR 'nursing home patient'/exp OR 'long term care'/de OR 'assisted living facility'/exp OR 'home for the aged'/exp OR ((nursing NEXT/2 home*) OR "skilled nursing facilit*" OR ("long term" NEXT/3 care) OR "care home*" OR "assisted living facilit*" OR ("assisted living" NEAR/2 resident*) OR "extended care facilit*" OR "intermediate care facilit*" OR "medical home*" OR (institutionali* NEXT/2 elderly) OR "geriatric homes" OR "home for the elderly" OR "homes for the elderly" OR "home for the aged" OR "homes for the aged"):ab,ti,kw) AND ('aged'/exp OR 'elderly care'/de OR 'geriatric care'/exp OR 'geriatric patient'/de OR 'geriatrics'/exp OR 'home for the aged'/exp OR (elder* OR eldest OR geriatr* OR "old age*" OR (older NEXT/1 (patient* OR people OR subject* OR age* OR adult* OR man OR men OR woman OR women OR population* OR person*)) OR aging OR ageing OR senior* OR "late life" OR "oldest old*" OR "very old*" OR "home for the aged" OR "homes for the aged" OR geronto* OR psychoger*)) AND ('falling'/exp OR 'fear of falling'/exp OR 'fall risk'/exp OR (fall OR falls OR falling):ab,ti,kw) AND ('mood disorder'/de OR 'depression'/exp OR 'antidepressant agent'/exp OR (depress* OR "mood disorder*" OR "affective disorder*" OR "mood decline" OR antidepress*):ab,ti,kw) NOT [conference abstract]/lim

**Medline ALL Ovid**

Ovid MEDLINE(R) ALL 1946 to October 26, 2023

397 references found, 27 October 2023

("Nursing Homes"/ OR "Long-Term Care"/ OR "Assisted Living Facilities"/ OR "Homes for the Aged"/ OR ((nursing ADJ2 home*) OR "skilled nursing facilit*" OR ("long term" ADJ3 care) OR "care home*" OR "assisted living facilit*" OR ("assisted living" ADJ2 resident*) OR "extended care facilit*" OR "intermediate care facilit*" OR "medical home*" OR (institutionali* ADJ2 elderly) OR "geriatric homes" OR "home for the elderly" OR "homes for the elderly" OR "home for the aged" OR "homes for the aged").ab,ti,kf.) AND (exp "Aged"/ OR "Geriatric Nursing"/ OR "Geriatrics"/ OR (elder* OR eldest OR geriatr* OR "old age*" OR (older ADJ1 (patient* OR people OR subject* OR age* OR adult* OR man OR men OR woman OR women OR population* OR person*)) OR aging OR ageing OR senior* OR "late life" OR "oldest old*" OR "very old*" OR "home for the aged" OR "homes for the aged" OR geronto* OR psychoger*).ab,ti,kf,jw,in.) AND ("Accidental Falls"/ OR (fall OR falls OR falling).ab,ti,kf.) AND ("Mood Disorders"/ OR exp "Depressive Disorder"/ OR "Depression"/ OR exp "Antidepressive Agents"/ OR (depress* OR "mood disorder*" OR "affective disorder*" OR "mood decline" OR antidepress*).ab,ti,kf.)

**CINAHL with Full Text EBSCO**

307 references found, 27 October 2023

(MH "Nursing Homes" OR MH "Nursing Home Patients" OR MH "Long Term Care" OR MH "Assisted Living" OR TI ((nursing W1 home*) OR "skilled nursing facilit*" OR ("long term" W2 care) OR "care home*" OR "assisted living facilit*" OR ("assisted living" N2 resident*) OR "extended care facilit*" OR "intermediate care facilit*" OR "medical home*" OR (institutionali* W1 elderly) OR "geriatric homes" OR "home for the elderly" OR "homes for the elderly" OR "home for the aged" OR "homes for the aged") OR AB ((nursing W1 home*) OR "skilled nursing facilit*" OR ("long term" W2 care) OR "care home*" OR "assisted living facilit*" OR ("assisted living" N2 resident*) OR "extended care facilit*" OR "intermediate care facilit*" OR "medical home*" OR (institutionali* W1 elderly) OR "geriatric homes" OR "home for the elderly" OR "homes for the elderly" OR "home for the aged" OR "homes for the aged")) AND (MH "Aged+" OR MH "Gerontologic Care" OR MH "Geriatrics" OR MH "Gerontologic Nursing+" OR TX (elder* OR eldest OR geriatr* OR "old age*" OR (older W1 (patient* OR people OR subject* OR age* OR adult* OR man OR men OR woman OR women OR population* OR person*)) OR aging OR ageing OR senior* OR "late life" OR "oldest old*" OR "very old*" OR "home for the aged" OR "homes for the aged" OR geronto* OR psychoger*)) AND (MH "Accidental Falls" OR TI (fall OR falls OR falling) OR AB (fall OR falls OR falling)) AND (MH "Affective Disorders+" OR MH "Antidepressive Agents+" OR TI (depress* OR "mood disorder*" OR "affective disorder*" OR "mood decline" OR antidepress*) OR AB (depress* OR "mood disorder*" OR "affective disorder*" OR "mood decline" OR antidepress*))

**APA PsycInfo Ovid**

APA PsycInfo 1806 to October Week 3 2023

180 references found, 27 October 2023

(exp nursing homes/ OR long term care/ OR assisted living/ OR ((nursing adj2 home*) OR "skilled nursing facilit*" OR ("long term" adj3 care) OR "care home*" OR "assisted living facilit*" OR ("assisted living" adj2 resident*) OR "extended care facilit*" OR "intermediate care facilit*" OR "medical home*" OR (institutionali* adj2 elderly) OR "geriatric homes" OR "home for the elderly" OR "homes for the elderly" OR "home for the aged" OR "homes for the aged").mp.) AND (geriatric patients/ OR exp geriatrics/ OR exp aging/ OR (380 OR 390).ag. OR (elder* OR eldest OR geriatr* OR "old age*" OR (older adj1 (patient* OR people OR subject* OR age* OR adult* OR man OR men OR woman OR women OR population* OR person*)) OR aging OR ageing OR senior* OR "late life" OR "oldest old*" OR "very old*" OR "home for the aged" OR "homes for the aged" OR geronto* OR psychoger*).mp,jw,in.) AND (falls/ OR (fall OR falls OR falling).mp.) AND (exp affective disorders/ OR "depression (emotion)"/ OR exp antidepressant drugs/ OR (depress* OR "mood disorder*" OR "affective disorder*" OR "mood decline" OR antidepress*).mp.)

**Cochrane Database of Systematic Reviews Wiley**

Cochrane Database of Systematic Reviews, Issue 10 of 12, October 2023

7 references found, 27 October 2023

((nursing NEXT home*) OR ("skilled nursing" NEXT facilit*) OR ("long term" NEXT care) OR (care NEXT home*) OR ("assisted living" NEXT facilit*) OR ("assisted living" NEAR resident*) OR ("extended care" NEXT facilit*) OR ("intermediate care" NEXT facilit*) OR (medical NEXT home*) OR (institutionali* NEXT elderly) OR "geriatric homes" OR "home for the elderly" OR "homes for the elderly" OR "home for the aged" OR "homes for the aged"):ab,ti,kw AND (elder* OR eldest OR geriatr* OR (old NEXT age*) OR (older NEXT (patient* OR people OR subject* OR age* OR adult* OR man OR men OR woman OR women OR population* OR person*)) OR aging OR ageing OR senior* OR "late life" OR (oldest NEXT old*) OR (very NEXT old*) OR "home for the aged" OR "homes for the aged" OR geronto* OR psychoger*) AND (fall OR falls OR falling):ab,ti,kw AND (depress* OR (mood NEXT disorder*) OR (affective NEXT disorder*) OR "mood decline" OR antidepress*):ab,ti,kw

**Cochrane Central Register of Controlled Trials Wiley**

Cochrane Central Register of Controlled Trials, Issue 10 of 12, October 2023

125 references found, 27 October 2023

((nursing NEXT home*) OR ("skilled nursing" NEXT facilit*) OR ("long term" NEXT care) OR (care NEXT home*) OR ("assisted living" NEXT facilit*) OR ("assisted living" NEAR resident*) OR ("extended care" NEXT facilit*) OR ("intermediate care" NEXT facilit*) OR (medical NEXT home*) OR (institutionali* NEXT elderly) OR "geriatric homes" OR "home for the elderly" OR "homes for the elderly" OR "home for the aged" OR "homes for the aged"):ab,ti,kw AND (elder* OR eldest OR geriatr* OR (old NEXT age*) OR (older NEXT (patient* OR people OR subject* OR age* OR adult* OR man OR men OR woman OR women OR population* OR person*)) OR aging OR ageing OR senior* OR "late life" OR (oldest NEXT old*) OR (very NEXT old*) OR "home for the aged" OR "homes for the aged" OR geronto* OR psychoger*) AND (fall OR falls OR falling):ab,ti,kw AND (depress* OR (mood NEXT disorder*) OR (affective NEXT disorder*) OR "mood decline" OR antidepress*):ab,ti,kw

**Web of Science Core Collection**

Science Citation Index Expanded (1900-present), Social Sciences Citation Index (1900-present), Arts & Humanities Citation Index (1975-present), Conference Proceedings Citation Index - Science (1990-present), Conference Proceedings Citation Index – Social Science & Humanities (1990-present), Book Citation Index - Science (2005-present), Book Citation Index – Social Sciences & Humanities (2005-present), Emerging Sources Citation Index (last 5 years), Current Chemical Reactions (1985-present), Index Chemicus (1993-present)

Advanced search > More options > Exact search

455 references found, 27 October 2023

TS=((("nursing" NEAR/1 home*) OR "skilled nursing facilit*" OR ("long term" NEAR/2 "care") OR "care home*" OR "assisted living facilit*" OR ("assisted living" NEAR/1 resident*) OR "extended care facilit*" OR "intermediate care facilit*" OR "medical home*" OR (institutionali* NEAR/1 "elderly") OR "geriatric homes" OR "home for the aged" OR "homes for the aged") AND (elder* OR "eldest" OR geriatr* OR "old age*" OR ("older" NEAR/1 (patient* OR "people" OR subject* OR age* OR adult* OR "man" OR "men" OR "woman" OR "women" OR population* OR person*)) OR "aging" OR "ageing" OR senior* OR "late life" OR "oldest old*" OR "very old*" OR "home for the elderly" OR "homes for the elderly" OR "home for the aged" OR "homes for the aged" OR geronto* OR psychoger*) AND ("fall" OR "falls" OR "falling") AND (depress* OR "mood disorder*" OR "affective disorder*" OR "mood decline" OR antidepress*))

**ProQuest Dissertations & Theses A&I**

Search options > Advanced search > Field : Anywhere except full text – NOFT > Doctoral dissertations only

23 references found, 27 October 2023

(fall OR falls OR falling) AND (depress* OR "mood disorders" OR "affective disorders" OR "mood decline" OR antidepress*) AND ("home for the aged" OR "homes for the aged" OR "nursing home" OR "nursing homes" OR "long term care" OR "assisted living")

Supplementary searches

**Google Scholar**

Search limits > Since 2017 > Do not include patents or citations

Fall|falls|falling depression "home for the aged"|"homes for the aged"|"nursing home"|"nursing homes"|"long term care facility"|"long term care facilities"|"assisted living facility"|"assisted living facilities"

**DART-Europe E-theses Portal**

(fall OR falls OR falling) AND (depress* OR "mood disorders" OR "affective disorders" OR "mood decline" OR antidepress*) AND ("home for the aged" OR "homes for the aged" OR "nursing home" OR "nursing homes" OR "long term care" OR "assisted living")

**ClinicalTrials.gov**

Advanced search

**Condition or disease** : depression

**Other terms** : (fall OR falls OR falling) AND ("home for the aged" OR "homes for the aged" OR "nursing home" OR "nursing homes" OR "long term care" OR "assisted living")

**Age group : older adult (65+)**

**ICTRP - WHO International Clinical Trials Registry Platform**

(fall OR falls OR falling) AND (home for the aged OR homes for the aged OR nursing home* OR long term care OR assisted living)
