# Supplementary material for: Relationship Between Depression and Falls Among Nursing Home Residents: Integrative Review
Source: Interact J Med Res. 2024 Nov 28;13:e57050. doi: 10.2196/57050 (PMC11638692; doi:10.2196/57050)
Supplement: Multimedia Appendix 2 [file ijmr_v13i1e57050_app2.docx]

Multimedia Appendix 2.

Detection and diagnosis of depression.

| **Study no** | **Authors** | **Scales of depression assessment** | **Categorisation** | **Assessment** |
| --- | --- | --- | --- | --- |
| 1 | Kioh, S. H. & Rashid, A. (2018). | 15-item Geriatric Depression Scale (GDS-15) | Score 0–5:  Score 6–8: Mild depression  Score 9–1: Moderate depression  Score 12–15: severe depression. | NR |
| 2 | Damian, J., Pastor-Barriuso, R., Valderrama-Gama, E. & de Pedro-Cuesta, J. (2013). | 10-item Geriatric Depression Scale (GDS-10) | Score 0–3: Normal  Score 4–7: Moderate  Score 8–10: Severe | Resident interviews concerning their status during the previous seven days. |
| 3 | Khater, M. S. & Mousa, S. M. (2012). | 15-item Geriatric Depression Scale (GDS-15) | NR | NR |
| 4 | Ku, Y. C., Liu, M. E., Tsai, Y. F., Liu, W. C., Lin, S. L. & Tsai, S. J. (2013). | 15-item Geriatric Depression Scale (GDS-15) | Score < 5 = without depression.  Score > 5 = with depression. | NR |
| 5 | Wang, Y. C., Lin, F. G., Yu, C. P., Tzeng, Y. M., Liang, C. K., Chang, Y. W., Chou, C. C., Chien, W. C. & Kao, S. (2012). | 15-item Geriatric Depression Scale (GDS-15) | Score < 5 = without depression.  Score > 5 = with depression. | Self-rated depressive symptoms. |
| 6 | Sylliaas, H., Selbaek, G. & Bergland, A. (2012). | Cornell Scale for Depression in Dementia | NR | Information from nursing home staff members. |
| 7 | Kron, M., Loy, S., Sturm, E., Nikolaus, T. & Becker, C. (2003). | Minimum Data Set (MDS) of the Resident Assessment Instrument (RAI), version 2.0. | NR | NR |
