# Supplementary material for: Relationship Between Depression and Falls Among Nursing Home Residents: Integrative Review
Source: Interact J Med Res. 2024 Nov 28;13:e57050. doi: 10.2196/57050 (PMC11638692; doi:10.2196/57050)
Supplement: Multimedia Appendix 3 [file ijmr_v13i1e57050_app3.docx]

Multimedia Appendix 3.

Definition and measurement of falls

| **Study no** | **Authors** | **Falls definition** | **Specificities** | **Method** | **Period** |
| --- | --- | --- | --- | --- | --- |
| 1 | Kioh, S. H. & Rashid, A. (2018). | Any incident that causes the person to rest on the floor unintentionally that occurred accidentally or non-accidentally. | NR | Questionnaire.  Fall Risk Assessment Tool. | 12 months before the questionnaire date. |
| 2 | Damian, J., Pastor-Barriuso, R., Valderrama-Gama, E. & de Pedro-Cuesta, J. (2013). | No specific definition. | NR | Interviews (physicians and nurses).  Medical records and nursing annotations.  Timed Up and Go test – falls risk assessment. | Month preceding the interview date. |
| 3 | Khater, M. S. & Mousa, S. M. (2012). | Any event in which a person inadvertently or unintentionally came to rest on the ground or a lower level, such as a chair, toilet, or bed (Delbaere K. et al., 2008). | NR | Events recorded during the study period. | |
| 4 | Ku, Y. C., Liu, M. E., Tsai, Y. F., Liu, W. C., Lin, S. L. & Tsai, S. J. (2013). | An event that results in a person coming to rest unintentionally on the ground or another lower level, but not due to any intentional movement, a major intrinsic event (e.g. stroke) or an extrinsic force (e.g. being forcefully pushed down, knocked down by a car, etc.) (Tinetti ME et al., 1998) | NR | Events recorded during the study period. | |
| 5 | Wang, Y. C., Lin, F. G., Yu, C. P., Tzeng, Y. M., Liang, C. K., Chang, Y. W., Chou, C. C., Chien, W. C. & Kao, S. (2012). | An unexpected event in which the person comes to rest on the ground, the floor or a lower level (Delbaere et al., 2010). | Faller = resident with two or more falls or with at least one injurious fall;  Non-faller: resident with a single fall or no falls. | Face-to-face interviews. | Prior one-year period. |
| 6 | Sylliaas, H., Selbaek, G. & Bergland, A. (2012). | An unexpected event in which the participant comes to rest on the ground, the floor or at a lower level (Lord et al., 2007). | RAS | Events recorded during the study period. | |
| 7 | Kron, M., Loy, S., Sturm, E., Nikolaus, T. & Becker, C. (2003). | Unintentionally coming to rest on the ground or at a lower level, regardless of losing consciousness. | Single and dual fallers are considered different from fallers with > 2 falls. | Events recorded during the study period. | |
