# Supplementary material for: Relationship Between Depression and Falls Among Nursing Home Residents: Integrative Review
Source: Interact J Med Res. 2024 Nov 28;13:e57050. doi: 10.2196/57050 (PMC11638692; doi:10.2196/57050)
Supplement: Multimedia Appendix 4 [file ijmr_v13i1e57050_app4.docx]

Multimedia Appendix 4.

Methodological quality of the retained cross-sectional studies according to the Appraisal tool for Cross-Sectional Studies (AXIS) tool (YN score)

| **Study** | **Selection** | | | | **Comparability** | | **Outcome** | | | **Total quality score** |
| --- | --- | --- | --- | --- | --- | --- | --- | --- | --- | --- |
|  | Representativeness of the exposed cohort | Selection of the non-exposed cohort | Ascertainment of exposure | Demonstration that the outcome of interest was not present at the start of the study | Adjustment for the most critical risk factors | Adjustment for other risk factors | Assessment of the outcome | Follow-up length | Loss to follow-up rate |  |
| Khater, M. S. & Mousa, S. M. (2012). | * | * | * | - | * | * | * | * | * | 8 |

Note: This study was evaluated on a scale from 0 to 9 stars. Good quality = (3 or 4 stars in the selection domain AND 1 or 2 stars in the comparability domain AND 2 or 3 stars in the outcome/exposure domain); Fair quality = (2 stars in the selection domain AND 1 or 2 stars in the comparability domain AND 2 or 3 stars in the outcome/exposure domain); and Poor quality = (0 or 1 star in the selection domain OR 0 stars in the comparability domain OR 0 or 1 stars in the outcome/exposure domain).
