# Supplementary material for: Relationship Between Depression and Falls Among Nursing Home Residents: Integrative Review
Source: Interact J Med Res. 2024 Nov 28;13:e57050. doi: 10.2196/57050 (PMC11638692; doi:10.2196/57050)
Supplement: Multimedia Appendix 5 [file ijmr_v13i1e57050_app5.docx]

Multimedia Appendix 5.

Methodological quality of the cohort study retained based on the Newcastle–Ottawa Scale guidelines

| **No** | **Studies** | **Intr** | **Methods** | | | | | | | | | | **Results** | | | | | **Discussion** | | **Other** | |
| --- | --- | --- | --- | --- | --- | --- | --- | --- | --- | --- | --- | --- | --- | --- | --- | --- | --- | --- | --- | --- | --- |
|  |  | **1** | **2** | **3** | **4** | **5** | **6** | **7** | **8** | **9** | **10** | **11** | **12** | **13** | **14** | **15** | **16** | **17** | **18** | **19** | **20** |
| 1 | Kioh, S. H. & Rashid, A. (2018). | Y | Y | Y | Y | Y | Y | N | Y | Y | Y | Y | Y | N | N | Y | Y | Y | Y | N | Y |
| 2 | Damian, J., Pastor-Barriuso, R., Valderrama-Gama, E. & de Pedro-Cuesta, J. (2013). | Y | Y | Y | Y | Y | Y | N | Y | Y | Y | Y | Y | N | N | Y | Y | Y | Y | N | Y |
| 4 | Ku, Y. C., Liu, M. E., Tsai, Y. F., Liu, W. C., Lin, S. L. & Tsai, S. J. (2013). | Y | Y | Y | Y | Y | Y | N | Y | Y | Y | Y | Y | N | N | Y | Y | Y | Y | N | Y |
| 5 | Wang, Y. C., Lin, F. G., Yu, C. P., Tzeng, Y. M., Liang, C. K., Chang, Y. W., Chou, C. C., Chien, W. C. & Kao, S. (2012). | Y | Y | Y | Y | Y | Y | N | Y | Y | Y | Y | Y | N | N | Y | Y | Y | Y | N | Y |
| 6 | Sylliaas, H., Selbaek, G. & Bergland, A. (2012). | Y | Y | N | Y | Y | Y | N | Y | Y | Y | Y | Y | N | N | Y | Y | Y | Y | NR | Y |
| 7 | Kron, M., Loy, S., Sturm, E., Nikolaus, T. & Becker, C. (2003). | Y | Y | N | Y | Y | Y | N | Y | Y | Y | Y | Y | N | N | Y | Y | Y | Y | NR | Y |

Note. 1 = Were the aims/objectives of the study clear?; 2 = Was the study design appropriate for the stated aim(s)?; 3 = Was the sample size justified?; 4 = Was the target/reference population clearly defined? (Is it clear who the research was about?); 5 = Was the sample frame taken from an appropriate population base so that it closely represented the target/reference population under investigation? 6 = Was the selection process likely to select subjects/participants representing the target/reference population under investigation? 7 = Were measures undertaken to address and categorise non-responders? 8 = Were the risk factors and outcome variables measured appropriate to the study’s aims?; 9 = Were the risk factor and outcome variables measured correctly using instruments/measurements that had been trialled, piloted or published previously?; 10 = Is it clear what was used to determine statistical significance and precision estimates? (e.g. *p*-values, confidence intervals); 11 = Were the methods (including statistical methods) sufficiently described to enable them to be repeated?; 12 = Were the basic data adequately described?; 13 = Does the response rate raise concerns about non-response bias?; 14 = If appropriate, was information about non-responders described?; 15 = Were the results internally consistent?; 16 = Were the results presented for all the analyses described in the methods?; 17 = Were the authors’ discussions and conclusions justified by the results?; 18 = Were the study's limitations discussed?; 19 = Were there any funding sources or conflicts of interest that may affect the authors’ interpretation of the results?; 20 = Was ethical approval or consent of participants attained?

Y = Yes; N = No; NR = Not reported
